# Climate change and marine cargo insurance - A global survey of insurers' perceptions

**DOI:** 10.1016/j.heliyon.2024.e37117

**Published:** 2024-08-31

**Authors:** Francois du Plessis, Leila Goedhals-Gerber, Joubert van Eeden

**Affiliations:** a Department of Logistics, Stellenbosch University, Private Bag X1, Matieland, 7602, South Africa; b Department of Industrial Engineering, Stellenbosch University, Private Bag X1, Matieland, 7602, South Africa

**Keywords:** Analysis of variance, Climate change, Marine cargo insurance, Perceptions, Ports, Supply chain risk management, Survey

## Abstract

The increasing frequency of climate-related hazards poses a significant risk to supply chains and marine insurance companies, which are already grappling with complex and interdependent global operations. Through a survey, this research examines the perceptions of an international cohort of marine insurers regarding their organization's participation in the Supply Chain Risk Management (SCRM) framework for climate change. In addition, the influence of respondents' experience levels and the World Bank's country classifications by income level are investigated. A repeated measures analysis of variance (ANOVA) is conducted to examine the effect of the SCRM framework's steps on perception, revealing significant variations among the steps and identifying gaps for improvement. While experience levels do not significantly affect involvement in the SCRM framework, distinct patterns emerge within each experience group, highlighting nuanced risk management practices. Comparing perceptions across World Bank income level categories reveals that higher country income levels generally correlate with higher average perception scores, indicating a potential association with greater awareness and management of climate change risks. The research also highlights the need for comprehensive involvement in all steps of the SCRM framework. Addressing climate change and building resilient supply chains requires a multi-faceted approach that includes enhanced risk management practices, and to this end, the authors' present areas for future research.

## Highlights

• Examines marine insurers in the Supply Chain Risk Management framework.
• ANOVA reveals variations in SCRM, indicating room for improvement.
• Experience doesn't sway SCRM participation, but alters risk practices.
• Higher income countries link to better climate change risk management.
• Advocates a diverse approach for resilient supply chains; notes research needs.

## Graphical abstract

**Figure:**
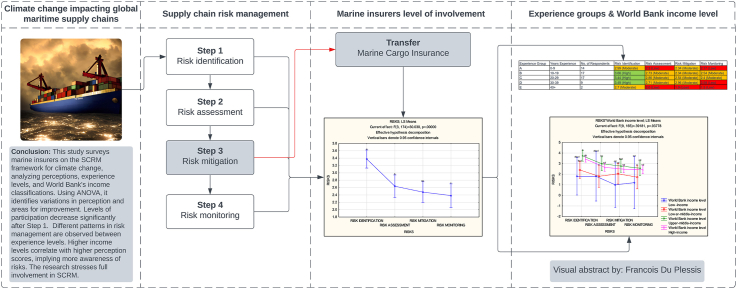

## 1 Introduction

One of humanity's most significant challenges is climate change, which has undeniable consequences for various aspects of our world, including physical, biological, human, and managed systems, with alarming future predictions [1]. A recent study by Kim et al. [2] published in Nature Communications aimed to improve climate models by incorporating satellite data from the past four decades [2]. The researchers used these adjusted models to project changes in Arctic ice under different greenhouse gas emission scenarios. Their findings indicate that an unprecedented ice-free Arctic climate could occur within the next 10–20 years, regardless of the emissions trajectory. In contrast, a study conducted by scientists at the Center for International Climate Research in Norway in 2020 suggested that the initial signs of global cooling would only emerge in approximately 25 years if annual emissions reductions of 5 % were implemented immediately [40]. However, the reality is that global emissions continue to rise [1].

According to the World Meteorological Organization [3], between 1970 and 2021, 11,778 reported disasters related to extreme weather, climate, and water events led to over two million deaths and economic losses, amounting to US$ 4.3 trillion. SwissRe, a prominent reinsurance company, estimated that the global economy suffered a loss of USD 190 billion due to natural disasters in 2020. Moreover, disaster-related losses (as a percentage of GDP) have increased by 1.6 % over the past 50 years based on a 10-year moving average [4], and this suggests that the same disaster today would cause greater damage compared to the past due to the growth in socio-economic value and other factors like climate change, thereby highlighting the potential impact of global warming on international trade [5].

Ocean shipping plays a crucial role in the supply chains and global trade of most industries, accounting for approximately 80 % (by volume) of goods transportation [6]. The volume of cargo transported by ships has significantly increased since 1990, more than doubling from four to nearly 10.7 billion tons in 2020 [6]. Correspondingly, the global merchant fleet has expanded its capacity by approximately 37 % from 2013 to 2020, reaching almost two billion deadweight tons in 2020, and increased by a further 5 % between 2020 and 2021 to reach 2.1 billion [6]. While cargo typically reaches its destination without issues, adverse events can occur during voyages. To mitigate this risk, cargo owners and supply chain risk managers can transfer the risk to marine cargo insurance companies [7,8].

Marine insurance covers the physical loss or damage of goods during transit and storage, from loading at the origin to unloading at the destination [9]. However, weather-related risks are inherently unstable and unpredictable, leading to high claims for insurers [10]. In addition, as companies (1) adopt new outsourcing models, (2) deal with shorter product cycles, (3) face high demand, and (4) operate globally, their supply chains become increasingly complex and interdependent [39]. This complexity exposes supply chains to higher vulnerability when faced with potential disruptions. Manners Bell [11] asserts that such intricate supply chains are particularly susceptible to external events, especially climate-related hazards that are becoming more frequent worldwide. Lam and Lassa [12] further highlight the heightened vulnerability of ports and shipping operations due to the increasing occurrence of natural hazards and extreme climate events.

While many insurers recognize the importance of building resilience to weather extremes, some question the necessity or feasibility of addressing the root causes of climate change or distinguishing between human and natural factors, and very little research on this topic currently exists [13]. Insurers adopt various strategies to cope with and transfer these risks, including exclusions of certain events from coverage or premium increases [14]. This context underlines the objectives of our research: The first aims to understand marine insurers' perceptions of their organizations' engagement in various stages of the Supply Chain Risk Management (SCRM) framework. The second explores whether the experience levels of the respondents correlate with their perceptions regarding this involvement. Lastly, the third question examines the influence of the World Bank's classification of countries by income level on these perceptions within the marine insurance sector.

In conclusion, climate change poses profound and far-reaching consequences for our world, with unsettling projections for the future. The possibility of an ice-free Arctic within the next two decades, irrespective of emissions, underscores the urgency of addressing this issue. The increasing frequency of extreme weather events continues to result in significant human and economic losses, amplifying the impact on global trade. Managing unpredictable weather risks remains a challenge for marine insurance, highlighting the significance of understanding the industry's perceptions and strategies in navigating these uncertainties.

The remaining structure of this article is organized as follows: Section 2 offers background information on climate change, its effects on ports and supply chains, and the theoretical concepts of marine insurance and supply chain risk management. Section 3 discusses the research methodology, followed by Section 4, which presents the results. Section 5 highlights the findings, while Section 6 provides implications for risk management, and Section 7 concludes the paper. Finally, in Section 8, potential areas for future research are proposed.

## 2 Literature review

Through a literature overview, this section provides the reader with a brief synopsis of this article's main topics, including (1) climate change, (2) its impacts on ports and supply chains, and finally, (3) marine cargo insurance and the theory of supply chain risk management. After reading this section, the reader should have a broad understanding of the topics and theory underpinning this article, which flows into the subsequent research questions.

### 2.1 Climate change

**Fig. 1:**
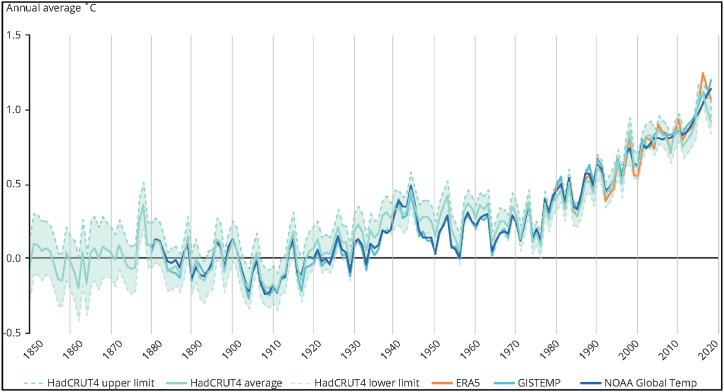
Global mean temperature rise (1850–2019).

Source: World Economic Forum [42].

According to the Intergovernmental Panel on Climate Change [1], global warming is primarily caused by human activities that release greenhouse gases into the atmosphere. The average global surface temperature has increased by 1.1 °C since the pre-industrial era (1850–1900), with a significant rise observed in the past decade (2011–2020) (refer to Fig. 1). The sources of greenhouse gas emissions vary across regions, countries, and individuals, depending on factors such as energy use, land use, lifestyles, and consumption patterns. These sources are not equally distributed or sustainable. The IPCC also highlights that human activities have resulted in warmer air, water, and land [1].

Recent data indicates a notable increase in global temperatures, with the five warmest years on record occurring since 2015, and 2020 being ranked as the second warmest year [15]. In addition, sea levels have risen by 200 mm from 1901 to 2018, with an acceleration observed in recent years [1]. Arctic sea-ice extent has decreased, and over the past two decades, ice sheet mass losses have surpassed gains [16]. Using satellite data, Smith et al. [16] reveal that Greenland is losing nearly 200 billion tonnes of grounded ice annually, while Antarctica is losing 118 billion tonnes annually, contributing to a 14-mm increase in global sea levels over the 16 years investigated (8.9 mm from Greenland and 5.2 mm from Antarctica). Human activities have been considered the primary driver of sea level rise since 1971, as well as an increased occurrence of extreme weather events such as heatwaves, heavy rain, droughts, and storms [1].

**Fig. 2:**
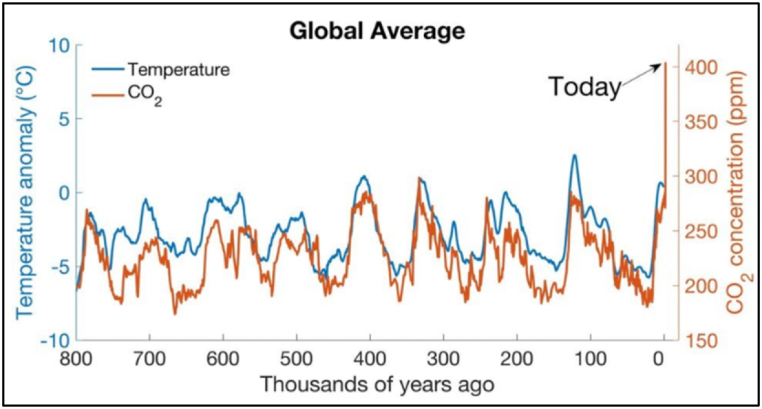
Temperature change (blue) and carbon dioxide change (brown).

Source: World Economic Forum (2019)

The rise in anthropogenic greenhouse gas emissions is predominantly attributed to the combustion of fossil fuels, which accounted for approximately 78 % of total CO2 emissions between 1970 and 2010 [17]. Past atmospheric observations from glacial cycles spanning over 800,000 years demonstrate an almost linear relationship between global temperature change and CO2 emissions [18]. Fig. 2 illustrates the relationship between temperature and carbon dioxide change over historical periods until 2019.

**Fig. 3:**
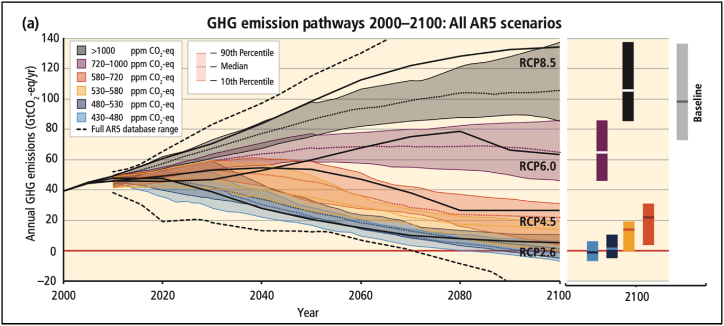
GHG emission pathways 2000-2100.

Source: IPCC [17].

To limit global warming to 1.5 °C with minimal overshoot, the IPCC's Fifth Assessment Report (AR5) indicates that global net anthropogenic CO2 emissions must reach net zero by 2050, accompanied by significant reductions in non-CO2 emissions [19]. The AR5 employs four Representative Concentration Pathways (RCPs) to project future greenhouse gas emissions until 2100, taking into account socio-economic development and climate policies (see Fig. 3, which provides an overview of greenhouse gas emission pathways) [17]. These pathways include an intense mitigation scenario (RCP2.6), two intermediary scenarios (RCP4.5 and RCP6.0), and a high-emission scenario (RCP8.5).

However, the latest synthesis report from the AR6 highlights that climate change poses higher risks than previously estimated in AR5 for any given level of future warming. The long-term consequences and losses associated with climate change are expected to be more severe than what is currently observed. The interaction between climate and non-climate risks will become more frequent, leading to complex and cascading risks that are increasingly challenging to manage [1]. In conclusion, the scientific evidence presented by the IPCC and other reputable sources highlights the alarming reality of climate change and its impacts on global systems. *It is important to note that for the purpose of this study, the AR5 RCPs were used for illustration, while AR6 employs a different Shared Socio-Economic Pathways (SSPs) illustration.*

### 2.2 Climate change impacts on ports and supply chains

The efficient operation of port cities is crucial for global economies due to the significant increase in seaborne trade, particularly in developing countries ([20,21]; Asariotis, Benamara & Mohos Naray, 2018). Ports play a vital role as critical nodes in the global supply chain, with approximately 80 % of trade volume and 70 % of trade value transported by sea [22]. Given the efficiency of maritime transportation, demands on port services and operations are projected to continue rising in the coming years [21].

However, ports are also highly vulnerable to the impacts of climate extremes due to their geographical location, as exemplified by the destruction caused by Hurricane Katrina in 2005, Sandy in 2012, Harvey in 2017, and Ida in 2021 [[20], [21], [22], [23]]. Climate change directly affects ports, including their infrastructure, services, and operations [22]. Recognizing the concern surrounding the impacts of climate change on maritime infrastructure, global organizations emphasize the necessity for proactive planning by ports to maintain their efficiency [21]. Thus, developing appropriate adaptation responses to mitigate these impacts is of strategic economic importance [22].

A survey conducted by Becker et al., in 2012, focusing on cargo handling ports worldwide, revealed that 47 % of the 342 surveyed ports were not currently planning for climate change. Many respondents expressed inadequate knowledge about climate change and lacked policies for adapting to its impacts. Insufficient long-term planning to mitigate extreme climate events and their implications for port cities was also noted by Hanson et al. [20]. The mismatch between the design lifetime of port infrastructure (30–50 years) and shorter capital planning cycles (5–10 years) was identified as a significant challenge hindering effective action [21].

The major climatic stressors affecting global ports include mean rise in sea levels, floods, storm surges, temperature extremes, heavy precipitation and fog, strong winds, and wave penetration [22]. Asariotis et al. [22] differentiate between direct impacts on ports, affecting operations, services, and infrastructure, and indirect impacts resulting from changes in demand for port services due to the impacts of climate change on various sectors.

Forecasting the future exposure of ports to climate extremes is crucial for informed decision-making and the development of adaptation strategies [24]. Hanson et al. [20] ranked the top 20 port cities in terms of assets exposed to coastal flooding from the present until the 2070s (see Table 1). They also identified various adaptation strategies, ranging from early warning systems and upgraded protection to land-use planning and risk-sharing through insurance and reinsurance.

**Table 1:** Cities ranked in terms of assets exposed to coastal flooding (projected until 2070).

Rank	Country	Urban Agglomeration	Exposed Assets — Current ($Billion)	Exposed Assets — Future ($Billion)
1	USA	Miami	416.29	3513.04
2	China	Guangzhou	84.17	3357.72
3	USA	New York-Newark	320.20	2147.35
4	India	Kolkata (Calcutta)	31.99	1961.44
5	China	Shanghai	72.86	1771.17
6	India	Mumbai	46.20	1598.05
7	China	Tianjin	29.62	1231.48
8	Japan	Tokyo	174.29	1207.07
9	China	Hong Kong	35.94	1163.89
10	Thailand	Bangkok	38.72	1117.54
11	China	Ningbo	9.26	1016.94
12	USA	New Orleans	233.69	1013.45
13	Japan	Osaka-Kobe	215.62	968.96
14	Netherlands	Amsterdam	128.33	843.70
15	Netherlands	Rotterdam	114.89	825.68
16	Vietnam	Ho Chi Minh City	26.86	652.82
17	Japan	Nagoya	109.22	623.42
18	China	Qingdao	2.72	601.59
19	USA	Virginia Beach	84.64	581.69
20	Egypt	Alexandria	28.46	563.28

The UNCTAD survey found that 69 % of respondents (which included port authorities, terminal operators, private ports, and port management companies) did not perceive observed climatic changes as a trend, except for some North American, European, and Asian ports. Furthermore, 42 % of respondents indicated a lack of corporate adaptation strategies, with a majority not developing such strategies. Insufficient availability of climatic parameter information, both past and present, was cited by many respondents as a barrier to identifying and planning port resilience and adaptation strategies [22].

### 2.3 Marine cargo insurance and supply chain risk management

In building resilience, insurers and reinsurers play a crucial role in promoting risk reduction strategies before the severe impacts of climate change affect port infrastructure and operations, ultimately impacting global supply chain efficiency [21].

However, despite the global scientific community's overwhelming consensus about climate change's future impact, the insurance industry has been slow to fully respond and adopt proactive measures and has been marked by a "wait and see" attitude [25]. Research conducted by the Chartered Insurance Institute as early as 1999 revealed this passive stance [25]. A study by Epstein and Mills in 2005 estimated that less than 1 % of insurance companies had adequately assessed the potential effects of climate change on their business operations [10]. To address this gap, regulatory bodies and regulators in the insurance sector established the Sustainable Insurance Forum (SIF) in 2016. SIF serves as a global collaborative platform to address sustainability issues, with climate change as a central focus [25].

**Fig. 4:**
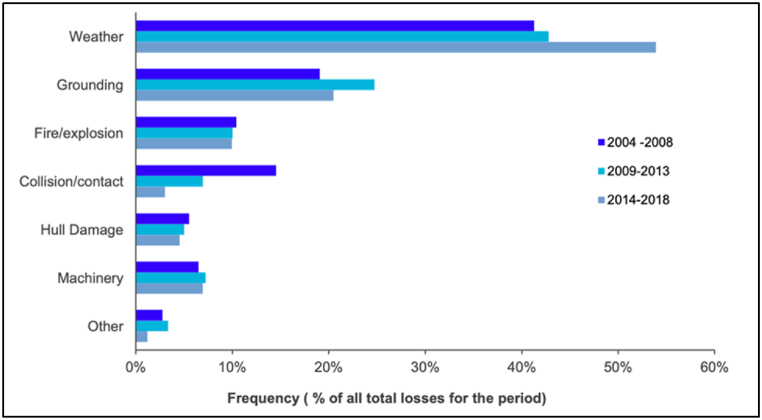
Frequency of losses, 2004–2018.

Source: IUMI [26].

Recent developments have further highlighted the urgency of addressing climate-related risks in the insurance industry. The International Union of Marine Insurance (IUMI) organized an industry-wide webinar that discussed the impact of climate change-induced extreme weather phenomena and the need to prepare for a "new normal" characterized by increased frequency of weather-related claims [26] (see Fig. 4). In a study conducted in 2023, du Plessis et al. found that weather-related marine cargo insurance claims have been on the rise over the past decade, leading to challenges and disruptions in the South African supply chain network. The study also highlighted that weather-related claims have higher average values and exhibit seasonal patterns compared to non-weather-related claims [27].

**Fig. 5:**
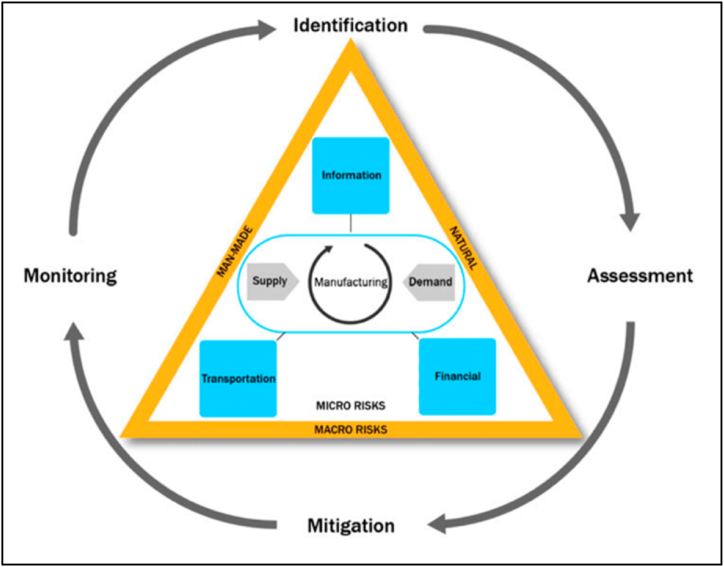
Supply chain risk management framework.

Source: Ho et al. [29].

The mounting complexity of maritime supply chains and the escalating risk exposure attributed to the potential effects of climate change necessitate a thorough review of risk exposure by insurance companies ([28]; [41]. Such assessments are critical as companies relying on marine cargo insurance for risk mitigation will experience adverse financial performance if they fail to account for these risks. Marine cargo insurance forms an essential component of the risk transfer strategies within the supply chain risk management (SCRM) framework proposed by Ho et al. [29] (see Fig. 5). The SCRM framework comprises four key steps: (1) identification, (2) assessment, (3) mitigation, and (4) monitoring [29]. Mitigation, as described by Tuncel and Alpan [30], involves reducing the severity of identified risks, which can be achieved through insurance coverage ([30]; du Plessis et al., 2023a).

According to a recent study conducted by Tarei et al. [31], the emphasis in existing research has primarily been on the identification of risks (step 1) and assessment of risks (step 2) within supply chains, while the implementation of strategies to minimize (step 3) and monitor (step 4) these risks has received limited attention (Tarei, Thakkar & Nag, 2020).

In conclusion, the traditional 'wait and see' approach adopted by the insurance industry towards climate change is no longer sufficient in effectively addressing the escalating risks linked to extreme weather events [25]. Research indicates a concerning rise in weather-related marine cargo insurance claims over the past decade, as evidenced by du Plessis et al. (2023a), posing significant challenges to the supply chain network and potentially jeopardizing the financial performance of companies. Given the trends mentioned above and their potential implications, understanding the perceptions of marine insurers becomes increasingly crucial.

## 3 Methodology

To the authors' knowledge, there is currently no literature that investigates the perceptions of marine insurers regarding the impacts of climate change. While studies have examined the perceptions of company managers on supply chain risks [32], researchers on supply chain risk management [33], and the public in Europe on climate change [38], the specific viewpoints of marine insurers remain unexplored. Therefore, building upon the research conducted by Tarei et al. [31], as discussed in Section 2.3, this article surveys marine cargo insurers' perceptions of climate change impacts using the steps outlined in the SCRM model from Section 2.3.

The research aims to address the following research questions (RQs) regarding the impacts of climate change on marine cargo insurance:

*RQ.*1 What is the perception of marine insurers regarding their organizations' level of participation in each step of the SCRM framework?

*RQ.2* Does the level of experience of respondents influence their perceptions?

*RQ.3* Does the World Bank's country classifications by income level correlate with perceptions?

The logical relationship among the three research questions is designed to explore marine insurers' perceptions within the Supply Chain Risk Management (SCRM) framework, aligning with the approaches of Tarei et al. [31] and Freichel et al. [34] for examining risk management practices. Initially, we establish a baseline of insurers' SCRM involvement, then investigate the impact of respondents' experience levels, and finally, assess the potential influence of external factors like the World Bank's country income classifications. This progression from internal to external influences allows for a nuanced understanding of risk management strategies across global contexts, mirroring the analytical depth of Freichel et al. [34] in examining how diverse factors shape the marine insurance industry's risk management approaches.

The research approach consists of three stages.

1. In the first stage (section 3.1), the focus is on developing, testing, and distributing the survey experiment.
2. The second stage (section 3.2) involves grouping the data and ensuring its reliability.
3. Finally, the third stage involves the analysis and presentation of the results, which are discussed in Section 4. A deeper discussion of the results follows in Sections 5, 6.

### 3.1 Developing, testing, and distributing the survey experiment

**Fig. 6:**
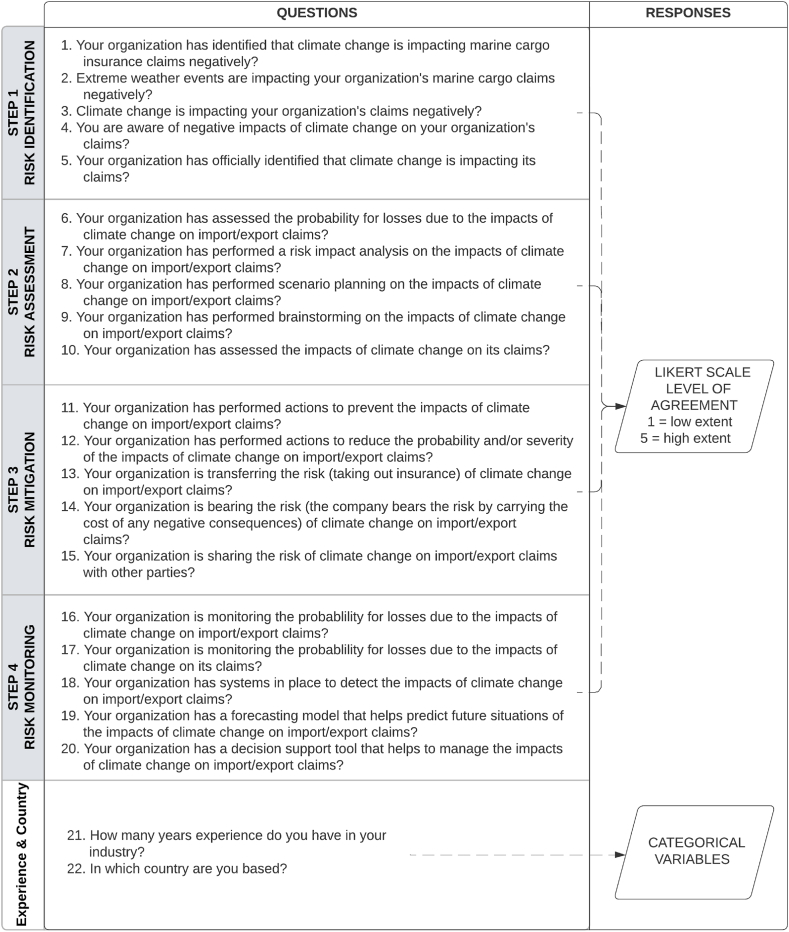
Survey experiment design.

The questionnaire development was based on a validated procedure utilized in previous research on supply chain operational risk perceptions conducted by Chen et al., in 2013 [35]. The questionnaire was adapted from their work to suit the specific objectives of this research. Initially, a literature review was conducted to identify pertinent constructs related to the research topic [36]. Based on the findings, a Likert scale questionnaire comprising 20 questions was developed to evaluate each step of the SCRM framework (refer to Fig. 6). It is important to note that all references to "marine cargo claims," "claims," and "import/export claims" in the survey pertain to the same concept of marine cargo insurance claims - which are handled by marine insurance companies.

Respondents were requested to express their agreement levels on a scale of 1 (low extent) to 5 (high extent), without being explicitly informed about the alignment of the questions with the different steps of the SCRM framework examined in the research. Furthermore, the questionnaire included additional questions to gather information about respondents' experience level in years (question 21) and country of operation (question 22).

A pilot test was conducted to ensure the questionnaire's appropriateness and effectiveness. Three industry experts and two researchers were selected for the pilot test. Their feedback and insights helped refine the questionnaire and ensure its relevance and reliability for data collection.

The survey received support from the International Union of Marine Insurance (IUMI), which facilitated its distribution to all global members via various digital platforms. IUMI, being the sole international organization dedicated solely to the marine insurance sector, typically includes hundreds of member organizations worldwide. As a global representative body for marine insurers, IUMI focuses on addressing industry-relevant matters. While specific membership statistics are not publicly disclosed, IUMI's broad coverage suggests substantial industry involvement. It's important to note that the views and insights shared primarily reflect the experiences and opinions of IUMI members rather than the entire marine insurance sector.

Research conducted received ethical clearance from the University of Stellenbosch, Social, Behavioural and Education Research Ethics Committee under project ID 24735. The survey was conducted online and was accessible for six months, starting from October 2022 and ending in March 2023 and informed consent was obtained from all participants. Throughout this timeframe, IUMI employed multiple methods to distribute the survey, including: (1) posting it twice on LinkedIn, (2) publishing an article in the IUMI Eye Newsletter with a link to the survey, and (3) informing participants about the survey during specific online training events. Unfortunately, due to the distribution methods used, it was not possible to accurately determine the exact number of IUMI members who received the survey. The survey received 59 responses from members in various countries (refer to Table 2). All surveys were fully completed. It is important to note that our research encountered difficulties in delineating sample populations, especially in regulated environments like financial services, where restrictions on information disclosure exist. These difficulties involved balancing individual perceptions with the representation of organizations. Although we obtained 59 responses, this sample size might be insufficient for making conclusive statements about variations among different groups. Furthermore, the limited number of responses reduces the ability to detect subtle differences and may limit the generalizability of the findings across broader populations.

**Table 2:** Country response frequency.

Country	Count	Percent
Australia	3	5 %
Canada	2	3 %
Egypt	2	3 %
France	3	5 %
Mexico	1	2 %
Germany	4	7 %
South Africa	7	12 %
Angola	1	2 %
India	2	3 %
Brazil	1	2 %
Japan	1	2 %
Belgium	4	7 %
Singapore	2	3 %
Austria	1	2 %
Malaysia	1	2 %
Sweden	13	22 %
Italy	1	2 %
Democratic Republic of the Congo	1	2 %
Netherlands	1	2 %
Poland	1	2 %
Colombia	1	2 %
United Kingdom	3	5 %
China	1	2 %
Chile	1	2 %
United States	1	2 %

The primary limitation of this study is the small sample size, which restricts the generalizability of the findings. While this limitation cannot be corrected in the current research, it is crucial to acknowledge its impact on the statistical analyses presented. The limited sample size may result in less robust conclusions, and caution should be exercised in interpreting the results. However, this limitation opens up numerous avenues for future research, where larger, more diverse samples could help validate and expand upon these initial findings.

### 3.2 Data grouping and reliability

**Fig. 7:**
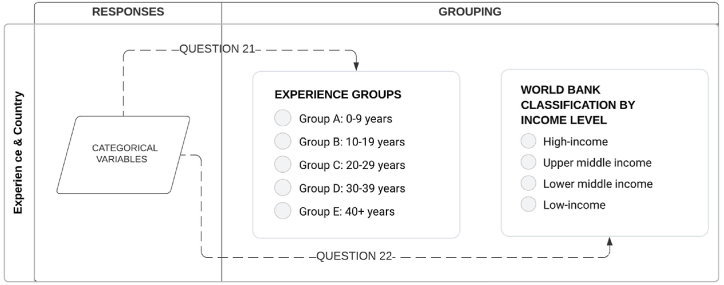
Data grouping.

To analyze the data obtained for questions 1 to 20 pertaining to the SCRM framework, Likert scale measurements were used, while questions 21 and 22 captured categorical variables. For statistical analysis, the data for questions 21 and 22 were subjected to grouping, as illustrated in Fig. 7.

The results from question 21 were analyzed and organized based on the respondents' experience levels, categorized in decade increments. There are 14 respondents with 0–9 years of experience in group A, 17 respondents with 10–19 years of experience in group B, 17 respondents with 20–29 years of experience in group C, 9 respondents with 30–39 years of experience in group D, and 2 respondents with over 40 years of experience in group E. As for question 22, the responses were grouped according to the World Bank's country classifications by income level for 2022–2023 [37]. The World Bank assigns countries to one of four income groups: low income, lower-middle income, upper-middle income, and high income. These classifications are updated annually on 1 July and are determined based on the previous year's gross national income (GNI) per capita, measured in USD. In the responses, there are 40 high-income respondents, 13 upper-middle-income respondents, 5 lower-middle-income respondents, and 1 low-income respondent.

The data reliability was assessed with the support of Stellenbosch University's Centre for Statistical Consultation, analyzing Cronbach's alpha and standardized alpha coefficients. The results indicate good to strong internal consistency across the constructs. 'Identification' showed a Cronbach's alpha of 0.812325 (standardized alpha: 0.812473), 'Assessment' had a Cronbach's alpha of 0.907370 (standardized alpha: 0.908103), 'Mitigation' exhibited acceptable internal consistency with a Cronbach's alpha of 0.741510 (standardized alpha: 0.748109), and 'Monitoring' demonstrated strong internal consistency with a Cronbach's alpha of 0.902135 (standardized alpha: 0.901364). These findings suggest consistent measurement of the intended concepts within each construct.

## 4 Analysis and results

The analysis and results for all the research questions are presented in this section, and with the assistance of Stellenbosch University's Centre for Statistical Consultation, Statistica 14® software was utilized for statistically analyzing the RQs. Section 5 provides a deeper discussion of the implications and interpretations of the findings.

### 4.1 RQ.1 what is the perception of marine insurers regarding their organizations' level of participation in each step of the SCRM framework?

A repeated measures analysis of variance (ANOVA) was used to test if the means of the different risks were the same. The dataset consisted of responses from a diverse group of (N = 59) marine insurers, encompassing individuals with varying experience levels and representing different global locations.

**Fig. 8:**
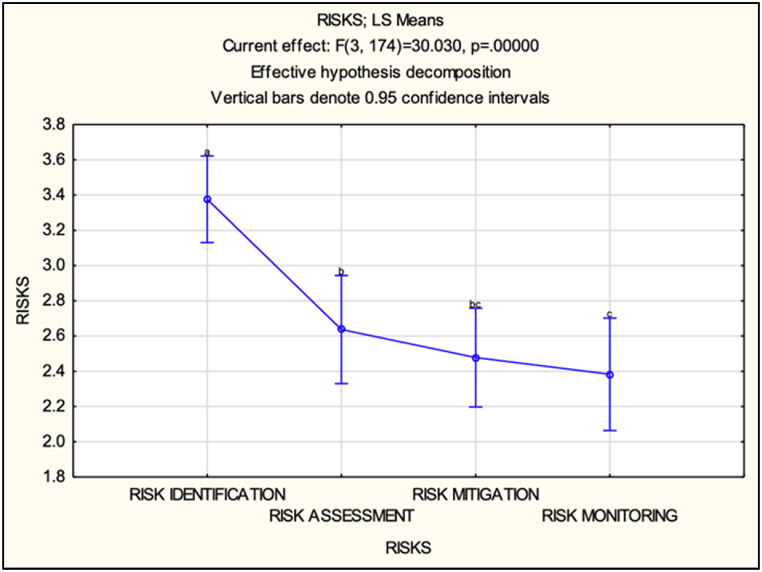
LS-means & post hoc groups (all respondents).

The findings from the repeated measures ANOVA revealed that the different ‘RISKS’ factor means differ significantly (F(3, 174) = 30.030, p < 0.001). The repeated measures on the same respondents were investigated using the compound symmetry assumption, i.e., equicorrelation over the four measures of risk. The subsequent LSD post hoc tests identified significant pairwise differences in perception between the steps of the SCRM framework. Specifically, ‘RISK IDENTIFICATION’ was perceived significantly differently from ‘RISK ASSESSMENT’ (p < 0.05) and ‘RISK MONITORING’ (p < 0.05). Moreover, ‘RISK ASSESSMENT’ was perceived significantly differently from ‘RISK MITIGATION’ (p < 0.05) and ‘RISK MONITORING’ (p < 0.05) (see Fig. 8 and Table 3).

**Table 3:** LSD test & post hoc groups (all respondents).

LSD test; variable DV_1						
Probabilities for Post Hoc Tests						
Error: Within MS = 0.39926, df = 174.00						
						
						
**Cell No.**	**RISKS**	**Group**	**{1} (3.3763)**	**{2} (2.6373)**	**{3} (2.478)**	**{4} (2.3831)**
1	RISK IDENTIFICATION	a	0	0	0	0
2	RISK ASSESSMENT	b	0	0	0.172611	0.030199
3	RISK MITIGATION	bc	0	0.172611	0	0.415692
4	RISK MONITORING	c	0	0.030199	0.415692	0

Overall, the findings imply that marine insurers' perceptions vary across the different steps of the SCRM framework. Specifically, the steps of risk identification differ significantly from the other risks (indicated by the letter a, while other letters are different from a), risk assessment and risk mitigation (letter b in common) do not differ, and risk monitoring (letter c) differs from risk identification and risk assessment (letters a and b). Thus, it can be observed from Fig. 8 that the steps of the SCRM framework are not uniformly perceived, and there is a noticeable decrease in levels of participation or effectiveness.

#### 4.1.1 RQ.2 Does the level of experience of respondents influence their perceptions?

To analyze this RQ, a repeated measures ANOVA was done by using the experience groups as fixed factor and with the assumption of compound symmetry over the repeated risk measures. The ANOVA table is given in Table 4.

**Table 4:** Repeated measures ANOVA (experience groups).

Effect	Repeated Measures Analysis of Variance Sigma-restricted parameterization Effective hypothesis decomposition				
	SS	Degr. of Freedom	MS	F	p
Intercept	849.3323	1	849.3323	222.7394	0
Experience group	10.7694	4	2.6924	0.7061	0.591275
Error	205.9085	54	3.8131		
RISKS	18.6955	3	6.2318	15.3179	0
RISKS*Experience group	3.5642	12	0.297	0.7301	0.720553
Error	65.907	162	0.4068		

**Fig. 9:**
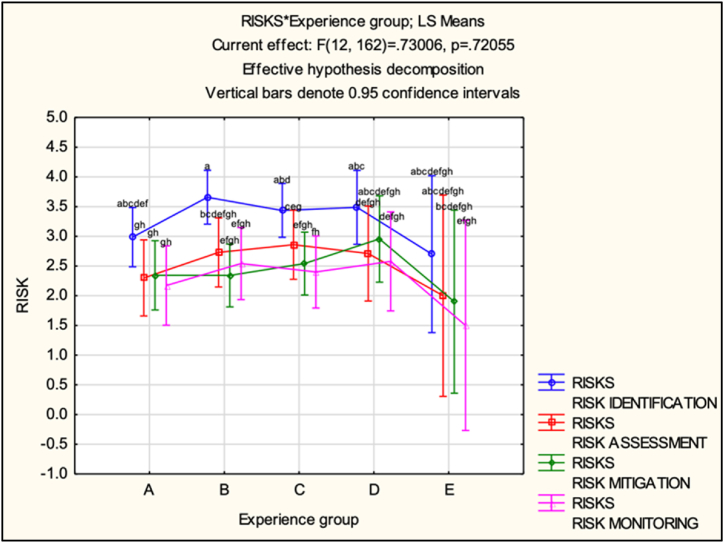
Interactions of 'RISKS' and 'Experience groups'.

The interaction between the factors 'RISKS' and 'Experience groups' is not statistically significant (F(12, 162) = 0.73, p = 0.7206). This suggests that the 'RISKS' means do not vary significantly across different levels of the 'Experience group.' In other words, the 'RISK' profiles (represented by the lines connecting different experience groups) appear relatively parallel, as shown in Fig. 9.

**Fig. 10:**
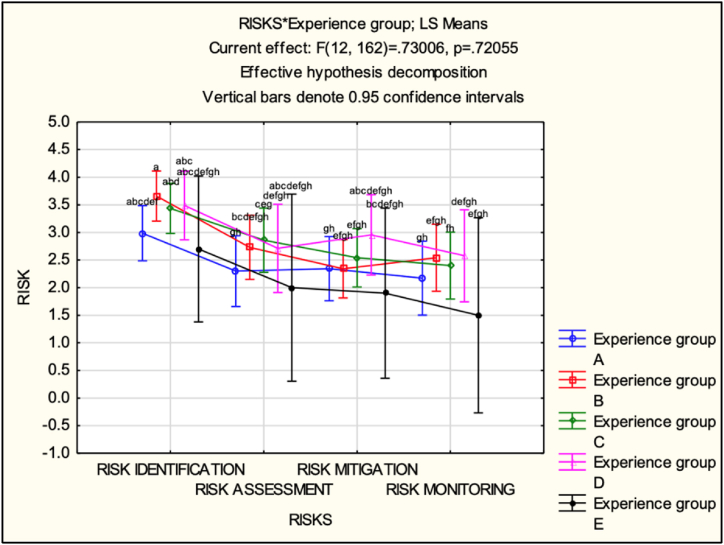
LS-means (experience groups).

Analyzing the LS-means data (see Fig. 10 and Table 5) provided insights into the respondents' perceptions within each experience group and step of SCRM. Regarding the perception of ‘RISK IDENTIFICATION’, respondents in group A (0–9 years) had a mean perception of 2.986 (SE = 0.249), while groups B (10–19 years), C (20–29 years) and D (30–39 years) exhibited higher mean perceptions ranging from 3.436 to 3.659. Group E (40+ years) had a lower mean perception of 2.7, albeit based on only two respondents. Regarding ‘RISK ASSESSMENT’, group A had the lowest mean perception at 2.3, whereas Group B had the highest at 2.729. Groups C and D displayed mean perceptions of around 2.859 and 2.711, respectively. Group E had a lower mean perception of 2.

**Table 5:** LS-means (experience groups).

Cell No.	RISKS*Experience group; LS Means Current effect: F(12, 162) = 0.73006, p = 0.72055 Effective hypothesis decomposition						
	Experience group	RISKS	Risk Mean	Risk Std.Err.	Risk -95,00 %	Risk 95,00 %	N
1	A	RISK IDENTIFICATION	2.985714	0.248982	2.486535	3.484893	14
2	A	RISK ASSESSMENT	2.3	0.319485	1.659471	2.940529	14
3	A	RISK MITIGATION	2.342857	0.290951	1.760258	2.925456	14
4	A	RISK MONITORING	2.171429	0.333225	1.242297	3.100562	14
5	B	RISK IDENTIFICATION	3.658824	0.225943	3.205828	4.11182	17
6	B	RISK ASSESSMENT	2.729412	0.289928	2.148142	3.310882	17
7	B	RISK MITIGATION	2.341176	0.263077	1.853571	2.828781	17
8	B	RISK MONITORING	2.541176	0.302397	1.934098	3.148255	17
9	C	RISK IDENTIFICATION	3.452941	0.292289	2.881308	4.024575	17
10	C	RISK ASSESSMENT	2.858824	0.289928	2.277553	3.440094	17
11	C	RISK MITIGATION	2.541176	0.302397	1.974376	3.107976	17
12	C	RISK MONITORING	2.7	0.306097	2.012476	3.387524	17
13	D	RISK IDENTIFICATION	3.488889	0.310535	2.866303	4.111476	9
14	D	RISK ASSESSMENT	2.711111	0.340982	2.055324	3.366899	9
15	D	RISK MITIGATION	2.955556	0.362043	2.229427	3.681684	9
16	D	RISK MONITORING	2.577778	0.415604	1.744541	3.411014	9
17	E	RISK IDENTIFICATION	2.7	0.658478	1.379296	4.020704	2
18	E	RISK ASSESSMENT	2	0.845278	0.305321	3.694679	2
19	E	RISK MITIGATION	1.9	0.688381	0.944133	3.855867	2
20	E	RISK MONITORING	1.5	0.88163	−0.26756	3.267562	2

Regarding ‘RISK MITIGATION’, groups A and D had similar mean perceptions of 2.343 and 2.956, respectively, while groups B and C showed slightly lower mean perceptions ranging from 2.341 to 2.541. Lastly, in terms of ‘RISK MONITORING’, group A had the lowest mean perception at 2.171, while group D had the highest at 2.578. Groups B and C had mean perceptions around 2.541 and 2.4, respectively, while group E had a lower mean perception of 1.5.

Based on the results, a heat map was created (see Table 6) for each experience group showing the level of perceived involvement within each step of the SCRM framework relating to climate change. The analysis reveals distinct patterns in the perceived risk management engagement levels among different experience groups.

**Table 6:**
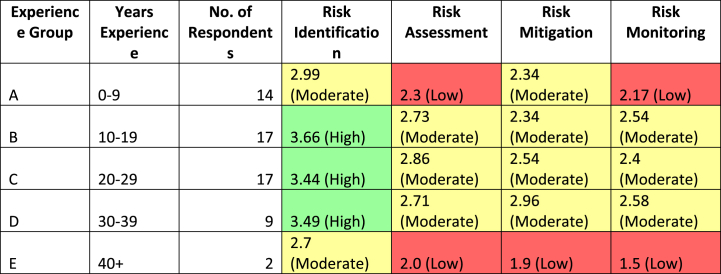
Heat map – experience groups.

In conclusion, the analysis of the LS-means data and corresponding heat map provided valuable insights into the risk perceptions (discussed in more detail in Section 5) within each experience group and across different steps of the SCRM framework.

#### 4.1.2 RQ.3 Does the World Bank's country classifications by income level affect perceptions?

This RQ investigates how the World Bank Country Classification correlates with perceptions of risks. A repeated measures ANOVA was conducted to analyze this, using the random factor 'RISKS' and the fixed factor 'World Bank income level.' This analysis follows a similar approach to the previous repeated measures ANOVA discussed in section 4.1.2, assuming compound symmetry across the levels of 'RISKS.'

The results of the repeated measures ANOVA demonstrate an interaction between the levels of 'RISK' and the 'World Bank income levels.' This interaction can be further interpreted using post-hoc LSD multiple comparisons. However, since this interaction is not statistically significant, the main effects of 'World Bank Income level' and 'RISKS' can be separately interpreted, similar to the discussion in section 4.1.1. The ANOVA table for this analysis is presented in Table 7.

**Table 7:** Repeated measures ANOVA (World Bank's country classifications by income level).

Effect	Repeated Measures Analysis of Variance (DATA 20230610) Sigma-restricted parameterization Effective hypothesis decomposition				
	SS	Degr. of Freedom	MS	F	p
Intercept	259.9211	1	259.9211	73.29648	0
World Bank income level	21.6391	3	7.213	2.03404	0.119738
Error	195.0388	55	3.5462		
RISKS	5.4394	3	1.8131	4.39835	0.005253
RISKS*World Bank income level	1.4536	9	0.1615	0.39181	0.937777
Error	68.0176	165	0.4122		

The results indicated levels of the main effect' World Bank income level' do not differ significantly, F(3,55) = 2.034 with p-value p = 0.1197.

**Fig. 11:**
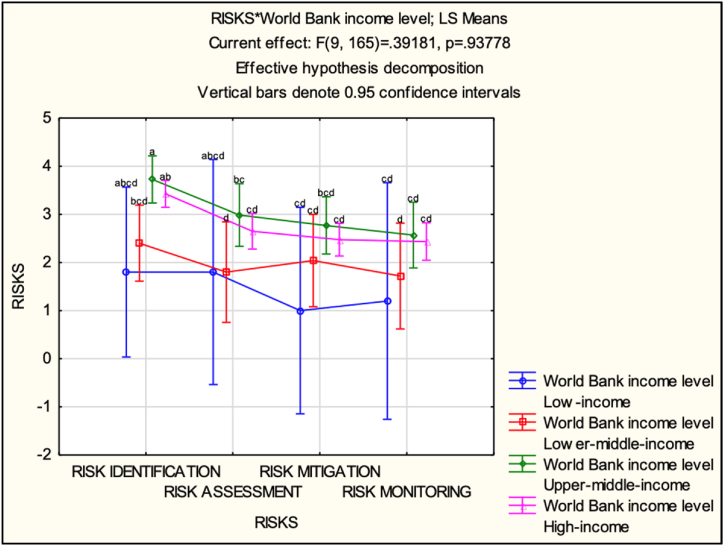
LS-means (World Bank's country classifications by income level).

LS means table and LSD post hoc multiple comparisons were made on the intersection means to compare the average perceptions across income levels and identify any patterns or differences (Table 8 and Fig. 11). The following patterns were observed (1) RISK IDENTIFICATION: the average perception score tends to increase as the income level rises. The highest average perception score is observed in the high-income category (3.425), followed by the upper-middle-income category (3.723077), lower-middle-income category (2.4), and low-income category (1.8). This suggests that countries with higher income levels may exhibit a greater awareness and identification of climate change risks; (2) RISK ASSESSMENT: similar to risk identification, the average perception score for risk assessment generally increases with higher income levels. The high-income category has the highest average perception score (2.65), followed by the upper-middle-income category (2.984615), lower-middle-income category (1.8), and low-income category (1.8). This indicates that countries with higher income levels might have a more comprehensive and accurate assessment of climate change risks; (3) RISK MITIGATION: the average perception score shows a mixed pattern. The lower-middle-income category has the highest average perception score (2.04), followed by the upper-middle-income category (2.769231), high-income category (2.475), and low-income category (1). This suggests that countries with middle-income levels might exhibit a stronger focus on climate change risk mitigation strategies; (4) RISK MONITORING: the average perception score also exhibits a mixed pattern. The upper-middle-income category has the highest average perception score (2.569231), followed by the high-income category (2.435), lower-middle-income category (1.72), and low-income category (1.2). This indicates that middle-to high-income countries may have more robust systems for monitoring and managing climate change risks.

**Table 8:** LS-means (World Bank's country classifications by income level).

Cell No.	RISKS*World Bank income level; LS Means Current effect: F(9, 165) = 0.39181, p = 0.93778 Effective hypothesis decomposition						
	World Bank income level	RISKS	DV_1 Mean	DV_1 Std.Err.	DV_1–95,00 %	DV_1 95,00 %	N
1	Low-income	RISK IDENTIFICATION	1.8	0.879443	0.03756	3.562443	1
2	Low-income	RISK ASSESSMENT	1.8	1.166322	−0.53736	4.137362	1
3	Low-income	RISK MITIGATION	1.8	1.068911	−1.14408	3.144078	1
4	Low-income	RISK MONITORING	1.9	1.226875	−1.25811	5.05811	1
5	Lower-middle-income	RISK IDENTIFICATION	2.4	0.939299	1.61181	3.188154	5
6	Lower-middle-income	RISK ASSESSMENT	2.3	0.521595	0.7547	2.8453	6
7	Lower-middle-income	RISK MITIGATION	2.04	0.478462	1.08114	2.99886	6
8	Lower-middle-income	RISK MONITORING	1.72	0.548541	0.6207	2.8193	7
9	Upper-middle-income	RISK IDENTIFICATION	3.723077	0.243914	3.24692	4.199231	13
10	Upper-middle-income	RISK ASSESSMENT	2.984615	0.32348	2.336385	3.632846	13
11	Upper-middle-income	RISK MITIGATION	2.769231	0.296278	2.17457	3.36389	13
12	Upper-middle-income	RISK MONITORING	2.569231	0.340191	1.88747	3.250991	13
13	High-income	RISK IDENTIFICATION	3.425	0.139052	3.146833	3.703167	40
14	High-income	RISK ASSESSMENT	2.65	0.148544	2.28043	3.01957	40
15	High-income	RISK MITIGATION	2.475	0.169162	2.13599	2.81401	40
16	High-income	RISK MONITORING	2.435	0.193938	2.04634	2.823661	40

A heat map has been generated and presented in Table 9 to depict the perceived engagement of each income group within the various stages of the Supply Chain Risk Management (SCRM) framework concerning climate change. This visual analysis uncovers clear variations in the engagement levels with risk management activities across the income groups, and is discussed in more detail in the subsequent sections.

**Table 9:**
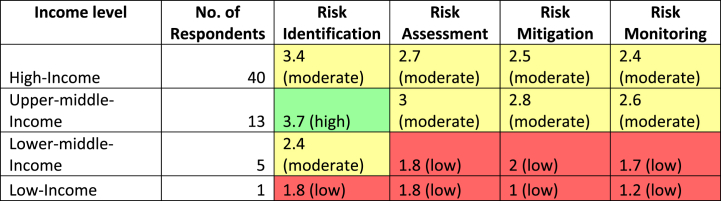
Heat map – World Bank's country classification by income level.

In summary, comparing the LS means across different World Bank income level categories allows one to observe patterns in average perceptions. Generally, higher income levels are associated with higher average perception scores, indicating a potentially greater awareness, assessment, and management of climate change risks. These inconsistent patterns indicate the presence of other influencing variables beyond income level in shaping perceptions. For example, a country's involvement with the United Nations Sustainable Development Goals (SDGs) could be a relevant factor, although it was not investigated in this research.

## 5 Discussion

This research aimed to examine the perception of marine insurers regarding their organizations' level of participation in each step of the Supply Chain Risk Management (SCRM) framework relating to climate change. In addition, the influence of respondents' experience levels and the World Bank's country classifications by income level on their perceptions were explored.

Firstly, the authors examined how marine insurers view their organizations' role in managing supply chain risks related to climate change. They used a framework that consists of four steps: identifying, assessing, mitigating, and monitoring risks. They applied a repeated measures analysis to test how the perception of involvement differed across these steps. They found significant variation, suggesting that some steps were more or less emphasized. They also detected gaps between the first step (identifying risks) and the others, implying room for improvement. This is consistent with previous research by Tarei et al. [31], who argued that existing studies have focused mainly on the first two steps while neglecting the last two [36].

Secondly, a repeated measures analysis of variance (ANOVA) was conducted to determine whether respondents' experience level influenced their overall perceptions. The analysis suggests that respondents' overall perceptions of 'RISKS' do not significantly differ based on their experience level. This finding highlights that factors beyond experience, such as organizational culture, management strategies, and technological adoption, may play more dominant roles in shaping individuals' perceptions. Future research could explore these factors in greater detail, as understanding these influences could offer new insights into effective risk management practices.

However, analyzing the LS-means data provided valuable insights into each experience group's perceptions and the SCRM framework's different steps. Different patterns in perceived risk management engagement levels emerged among different experience groups, shedding light on the nuanced dynamics of risk management practices within marine insurance organizations. Participants in Group B, with 10–19 years of experience, demonstrated heightened proficiency in risk identification and assessment, indicating their effective recognition and evaluation of potential risks. They also displayed moderate engagement in risk mitigation and monitoring, suggesting a proactive approach to managing identified risks. Similarly, Group C participants, with 20–29 years of experience, exhibited elevated risk identification and assessment capabilities, along with moderate mitigation and monitoring involvement.

Conversely, Group D participants (30–39 years of experience) showcased significant expertise in risk identification and moderate involvement in assessment and mitigation. In contrast, Group E individuals (40+ years of experience) exhibited moderate engagement across all risk management domains. It is important to note that the generalizability of the findings for Group E may be limited due to the small sample size of only two respondents. This limitation underscores the need for future studies to include larger and more diverse samples, which could help validate and expand upon these findings, providing more robust conclusions.

Lastly, the authors examined the influence of the World Bank's country classifications by income level on perceptions. LS means across different World Bank income level categories was compared to gain insights into average perceptions. Overall, higher income levels are generally associated with higher average perception scores, suggesting a potential correlation with greater awareness, assessment, and management of climate change risks. However, it is important to note that the patterns observed are inconsistent across all dependent variables. This inconsistency indicates that factors other than country income level, such as regional regulations, access to technological resources, and collaboration among industry stakeholders, may also significantly shape perceptions. Future research could further explore these dimensions, contributing to a more comprehensive understanding of risk management practices within the marine insurance sector.

In summary, this study validates expected outcomes within the marine insurance and climate change context, revealing nuanced dynamics and suggesting new research and practical applications. Its global scope and use of the SCRM framework offer unique insights, particularly as it is the first to explore marine insurers' perceptions, a previously neglected area. Building on du Plessis et al. [36], it fills a critical gap and provides a fresh perspective on marine insurance risk management literature.

## 6 Risk management implications

The survey on marine insurers' perceptions highlights critical risk management implications. Firstly, the varied recognition and involvement in the Supply Chain Risk Management (SCRM) framework's steps underscore the necessity for a more integrated approach. Insurers must enhance their identification, assessment, mitigation, and monitoring of climate change risks to ensure comprehensive coverage and proactive risk management.

Secondly, the findings suggest that experience levels among marine insurers do not significantly influence the perception of risk, indicating a universal need for education and training across all levels of expertise. This uniformity calls for industry-wide standards and practices that can be adopted to improve resilience against climate-related hazards.

The disparity in risk management practices across different income levels suggests that higher-income countries have a better grasp of climate change risks, possibly due to more resources and access to information. This disparity underscores the importance of global cooperation and knowledge sharing to elevate risk management practices in lower-income countries, making them more resilient to climate change impacts.

Finally, the research points to an urgent need for innovation in insurance services that can address the evolving nature of climate change risks. This need for innovation is derived from the findings related to the perception of marine insurers regarding their organizations' level of participation in each step of the SCRM framework. The significant variations in how insurers perceive their involvement in different steps, particularly in areas such as ‘RISK IDENTIFICATION’ and ‘RISK MONITORING,’ highlight gaps that can be addressed through more sophisticated risk assessment tools and models. These tools would enable insurers to predict future climate scenarios more accurately, allowing for better pricing and risk management strategies.

## 7 Conclusions

Climate change poses one of the most significant challenges to humanity, with far-reaching consequences for various aspects of our world. The alarming predictions and research findings discussed in this article emphasize the urgent need for action. The incorporation of satellite data in climate models, as demonstrated by Kim et al. [2], has provided valuable insights into the potential future of the Arctic ice. Regardless of the greenhouse gas emissions trajectory, an ice-free Arctic climate could become a reality within the next 10–20 years, highlighting the severity of the situation.

While some studies suggest the potential for global cooling to emerge in the coming decades through emissions reductions, the reality remains that global emissions continue to rise. The increasing frequency of extreme weather events and their devastating impacts, as reported by the World Meteorological Organization, underscores the urgency of addressing climate change. The economic losses and human lives affected by these disasters demonstrate the need for proactive measures.

The role of ocean shipping in global trade cannot be understated, as it accounts by volume for approximately 80 % of goods transportation. The growth in cargo volume and the expansion of the global merchant fleet further emphasize the importance of supply chain resilience in the face of potential disruptions. Marine insurance is crucial in mitigating risks associated with weather-related events during transit and storage. However, insurers face challenges due to the inherent unpredictability of weather-related risks and the increasing complexity of supply chains.

The research discussed in this article explores the perceptions of marine insurers regarding their organizations' involvement in the Supply Chain Risk Management (SCRM) framework, specifically in the context of climate change. The findings highlight the variation in perception among different steps of the SCRM framework, pointing to areas for improvement and the need for more comprehensive involvement in all the framework steps. While respondents' experience levels did not significantly affect involvement in the SCRM framework, distinct patterns emerged within each experience group, showcasing nuanced dynamics of risk management practices related to the SCRM framework's individual steps.

In addition, the findings on the World Bank's country classifications by income level highlight the influence of income levels on the average perceptions of climate change risks. Higher-income countries tend to demonstrate a greater awareness, assessment, and monitoring of climate change risks. Additionally, middle-income countries appear to prioritize risk mitigation efforts more strongly. These observations emphasize the importance of economic factors in shaping countries' perceptions and responses to climate change. By understanding the patterns and differences in perceptions across income levels, targeted interventions and policies can be developed to enhance SCRM efforts, particularly in lower-income countries where awareness and resources may be limited.

While the sample size in this study was relatively small, it provided valuable insights and a solid foundation for understanding the key trends and patterns in the data. Although a larger sample could further enhance the robustness of the findings, the results obtained here still offer meaningful contributions to the field. Future research with expanded sample sizes will build on these initial findings, helping to confirm and extend the conclusions drawn in this study.

In conclusion, addressing climate change and its impact on international trade requires a multi-faceted approach. Efforts to reduce global emissions must be prioritized to mitigate the severity of climate-related disasters. Moreover, building supply chain resilience and enhancing risk management practices within marine insurance organizations are crucial for navigating global supply chains' increasing complexity and vulnerability. Future research should delve deeper into these areas to inform effective strategies and policies that can mitigate the risks associated with climate change and ensure a sustainable future for international maritime trade.

## Future work

This research provides valuable insights into the perception of marine insurers regarding their organizations' level of participation in the Supply Chain Risk Management (SCRM) framework for climate change. Building upon these findings, future research can further explore several areas to advance our understanding of risk management practices and inform effective strategies for enhancing engagement within the marine insurance industry.

1. Examining the impact of organizational culture and management strategies: This research touched upon the influence of factors beyond experience level on perceptions, suggesting that organizational culture and management strategies may play a significant role. Future research can delve deeper into the specific aspects of organizational culture that affect risk management engagement. This can involve exploring leadership styles, communication channels, decision-making processes, and the climate for risk awareness and response within marine insurance organizations.
2. Assessing the impact of regulatory frameworks: Regulatory frameworks and compliance requirements can significantly shape risk management practices within the marine insurance industry. Future research can investigate how existing regulations, such as those related to climate change mitigation and adaptation, influence insurers' perceptions and engagement in the SCRM framework. In addition, exploring the effectiveness of regulatory mechanisms in promoting sustainable risk management practices can provide insights for policymakers and industry stakeholders.
3. Investigating industry collaboration: Supply chain risks related to climate change are complex and interconnected. Collaboration among various stakeholders, including insurers, shippers, suppliers, and regulators, is crucial for effective risk management. Future research can explore collaborative initiatives and partnerships within the marine insurance industry, examining their impact on risk management practices and identifying best practices for fostering collaboration and knowledge sharing.
4. Extending the research to other industry sectors: While this research focused on marine insurers, the SCRM framework and its relevance to climate change apply to various industry sectors. Future research can extend the investigation to other sectors, such as logistics, manufacturing, and transportation, to better understand risk management perceptions and practices. Comparing and contrasting risk management approaches across industries can facilitate cross-learning and the development of comprehensive risk management strategies.
5. Enhancing sample size in future studies: While the current study's sample size is modest, future research presents an opportunity to build on these findings by incorporating a larger and more diverse group of marine insurers. Expanding the sample, potentially by engaging participants at industry events, would enable more robust statistical analyses and could reveal variations among different groups that were not captured in this study. A larger sample size would also strengthen the identification of trends, validate the present findings, and contribute to the development of more targeted risk management strategies.
6. In addition to the current findings, future research could focus on developing a model to forecast the effects of changing weather-related events on claims within the marine insurance industry. Such a model would provide valuable data to assist stakeholders, including marine cargo insurers and cargo owners, in making strategic risk-related decisions.

Addressing these areas of future research will help to advance our understanding of risk management practices, identify areas for improvement, and develop effective strategies for enhancing engagement in the SCRM framework.

## Funding

This research did not receive any specific grant from funding agencies in the public, commercial, or not-for-profit sectors.

## Ethical considerations

Ethical clearance to conduct the research was obtained from the University of Stellenbosch, Social, Behavioural and Education Research Ethics Committee (project ID 24735).

## Data availability statement

Data will be made available upon request.

## CRediT authorship contribution statement

**Francois du Plessis:** Writing – original draft, Visualization, Validation, Methodology, Investigation, Formal analysis, Conceptualization. **Leila Goedhals-Gerber:** Writing – review & editing, Validation, Supervision. **Joubert van Eeden:** Writing – review & editing, Validation, Supervision.

## Declaration of competing interest

To whom it may concern:

The authors of the article titled “CLIMATE CHANGE AND MARINE CARGO INSURANCE - A GLOBAL SURVEY OF INSURERS' PERCEPTIONS” hereby confirm that they have **NO CONFLICTS OF INTEREST.**
